# The Expansion of Artificial Intelligence in Modifying and Enhancing the Current Management of Abdominal Aortic Aneurysms: A Literature Review

**DOI:** 10.7759/cureus.66398

**Published:** 2024-08-07

**Authors:** Usman Khalid, Hristo A Stoev, Boyko Yavorov, Areeb Ansari

**Affiliations:** 1 Medicine, Medical University of Plovdiv, Plovdiv, BGR; 2 Cardiac Surgery, St. George University Hospital, Plovdiv, BGR; 3 Cardiovascular Surgery, Medical University of Plovdiv, Plovdiv, BGR

**Keywords:** abdominal aortic aneurysms, neural network, diagnostics, vascular surgery, artificial intelligence in medicine

## Abstract

An abdominal aortic aneurysm (AAA) is a pathological dilation that is 3 cm or greater resulting in a bulging or balloon appearance. To meet a personalized therapeutic approach for patients, artificial intelligence (AI) can exhibit an array of applications ranging from decoding patterns from large data sets to predicting new data. The review aims to discuss how AI can assist and improve the standard of care and management plans for these patients. A comprehensive non-systematic literature review was carried out for published material on the use of AI relating to AAAs. The PubMed and Google Scholar databases were used to scout for articles relating to the title of this review. The review included 54 literature papers in this study. AI is involved on a genomic level, which assists in screening, diagnosing, and identifying individual risk factors of a patient. Personalized management plans can be created with AI predictions using patient data to reduce the risk of in-hospital mortality following a repair or due to complications. AI represents a promising group of programs aimed at improving patient management and assisting surgeons in making beneficial decisions to improve the patient’s prognosis.

## Introduction and background

An abdominal aortic aneurysm (AAA) is a pathological dilation that is 3 cm or greater, which occurs when a weakness in the wall causes a bulging or balloon appearance. A progression to focal dilation can predispose the abdominal aorta to rupture once the dilation grows beyond 5.5 cm [1]. When managing patients with AAA according to the guidelines of the European Society for Vascular Surgery, evaluating the balance between the risk of AAA rupture and the surgical risk is key [2]. To formulate a personalized therapeutic approach for patients, AI can display an array of applications, from understanding patterns within large data sets to predicting new data and genomic analysis [3]. The complexities of AI build the foundation for new approaches to diagnosis, prognosis, and therapy tailored for individual patients. The difficult prediction of a rupture or complications following a repair makes a surgical approach challenging. These constraints could be alleviated through predictive machine learning (ML), which incorporates individual patient data as variables creating an accurate outcome using adaptive algorithms [4]. The review aims to discuss how artificial intelligence (AI) can improve and enhance the management of patients with AAA.

## Review

Methods

A comprehensive non-systematic literature review was carried out for published material on the use of AI relating to AAAs. The research was gathered primarily but not limited to PubMed and Google Scholar as the major databases to scout articles relating to the combination of the following terms: “abdominal aortic aneurysm,” “artificial intelligence,” “artificial neural network,” and “machine learning.” All materials published before 22 June 2024 were acceptable sources for this review. Articles that were not published in English were not included in this literature review.

Genomic ML

ML utilizes specific approaches to identify biomarkers using random forest (RF) [5]. RF is a non-parametric classification method that isolates specific biomarker data according to decision trees. This allows data separation and categorization to predict biomarkers with outstanding accuracy [6]. RF identified two biospecific markers used to distinguish between large/small AAAs and dilated AAAs. G0S2 exhibited significant upregulation in AAA tissues, and heparinase (HPSE) showed significant upregulation in small AAA samples. ROC curve analysis further underscored G0S2’s capability to accurately diagnose large AAAs. By assessing these biomarkers, health management can focus specifically on controlling the aneurysm and preventing its rupture by creating a new therapeutic and diagnostic reference point relating to the size of the dilation [5]. An individual study created HEAL (hierarchical estimate from agnostic learning) from ML, which successfully identified disease-associated components by grouping data from population genomes. When combined with health records, it displayed the relationship between the individuals’ baseline genome and their lifestyle choices, which can influence AAA predisposition, presenting its potential as a personal health management tool [7]. Genomic-inspired predictive AI can be valuable for developing clinical tests that target disease prediction and lead to improved prevention.

Prediction of AAA growth

ML has paved a path to predict outcomes from individual data sets of a patient enabling the creation of personalized health management plans for patients with an AAA. It uses the baseline flow-mediated dilation (FMD) and the AAA diameter. Using these figures and following benchmark techniques involving non-linear kernel SVR, the kernel allows the AI to understand non-linear patterns between FMD and AAA diameter allowing concise conclusions to be drawn despite being presented with complexified patterns [8]. It groups data processing, interference, and model evaluation creating an accurate prediction model [9]. Through these ML techniques, the AI successfully predicted individual AAAs within 2 mm in 85% and 71% of patients at 12 and 24 months, respectively [8]. The development of an accurate predictor of AAAs would revolutionize the way we manage patients with AAA. It would minimize premature screenings and surveillance scans relieving economic burdens by fractioning the total number of scans carried out. The patient outlook is also boosted as having a prediction of growth would allow for a streamlined health plan concerning lifestyle balance and planned surgical interventions, which can alleviate patients' concerns and worries. The confluence of AI-assisted prediction with personalized management plans has a promising outlook on the patient's overall care and quality of life.

Predicting in-hospital mortality following repair of AAA

A rising number of AI tools are available to explore and predict hospital mortality after repair or ruptured AAA. Artificial neural networks (ANNs) are AI programs designed to model a data set where it is taught to interlink and solve complex relationships between variables and create predicted outcomes [10]. Reintervention is another risk that must be assessed by surgeons following repair, and difficulties can arise due to the unpredictable nature of an aneurysm [11]. Through ML, using common preoperative data, the AI was able to categorize patients’ five-year risk from both endograft complications and all-cause mortality [12]. Similarly, ML programs are using Bayesian networks (BN) to predict hospital mortality in patients undergoing open repair of AAA. These networks can work with incomplete data sets and determine the probability of a specific outcome associated with the likelihood of patient survival after surgery. When the computational complexity is reduced, BN reaches an accuracy of 96.1%, a sensitivity of 86.8%, and a specificity of 96.8% [13]. In alternative studies where the intelligent network was compared to clinicians, ANN models outcompeted clinicians for the prediction of mortality following AAA repair [14]. These predictive networks could assist the surgeon in carrying out a personalized risk assessment when dealing with patients at different stages of their AAA. By evaluating the impact of interventions and treatment, before, during, or after the post-operative stage, the resource management could be optimized and distributed on a case-to-case basis to reduce total mortalities from AAA. AI can supplement the learning of trainee clinicians by giving them a more informed prognosis and sharpening their clinical judgment for patient management.

Currently, surveillance after the endovascular repair is based on the incidence of endograft complications; however, with prediction intelligence, the patient can be assessed individually and follow a specific care plan, which would prevent mortality due to unexpected complications. It has been documented that most re-interventions due to complications arise from symptoms, rather than periodic surveillance [15]. The number of unnecessary surveillance scans could be as high as 90% with 15% of patients experiencing complications despite normal findings on the scans [16]. Comparatively speaking, a predictive AI would not only prevent the number of scans that can be reallocated for other patients but it would allow for timelier surgical intervention reducing mortality and essentially allowing the surgeon to devise intervention plans curated to the patient's situation [17].

Automated detection of AAA

AI algorithms are essential for the automated detection of AAA through the analysis of various medical imaging scans, including CT scans, MRIs, and ultrasounds. AI is becoming commonly utilized in the evaluation and care of AAA. Studies have shown that AI enhances image segmentation enabling automated assessments and morphological descriptions of AAA. This disease is life-threatening with treatment being limited to open or endovascular surgery. Evaluating the need for surgery has been difficult in the clinical setting, and there is hope that AI may provide new insights and assist in the evaluation and risk assessment [4]. In the realm of CT scans, AI has displayed sensitivity and specificity, in identifying AAA, boasting a sensitivity of 95% and specificity of 96.6% [18]. Similarly, in the detection of acute findings in abdominal CT scans, an AI-based system achieved a sensitivity of 93% and specificity of 97% [19]. A recently developed deep learning technique that can automatically detect and evaluate the maximum aortic diameter is augmented radiology for vascular aneurysms (ARVR). It utilizes pre- and post-operative contrast-enhanced computed tomography angiography (CTA) data, allowing it to measure the exterior diameter of the aortic wall from the ascending aorta to the iliac bifurcations with accuracy, increasing the early detection of AAA. This automatic method provides a potentially reliable AI tool to assist clinical practice [20]. However, the AortaScan, a 3D ultrasound tool that automatically gauges the diameter of the abdominal aorta has been noted to have lower levels of sensitivity and specificity of 81% when compared with CT imaging. The AortaScan can identify AAA without the assistance of a trained operator, but more technological advancement is required to boost sensitivity [21]. AI can also analyze hazard analysis reports and observations to provide insights on conditions, activities, incident causes, and measures, for risk reduction [22]. The potential for AAA screening during echocardiography has been demonstrated, highlighting its viability, and indicating room for enhancement through AI technology [23]. Using AI in imaging has the potential to significantly enhance the accuracy and efficiency of AAA screening compared to traditional methods.

DSS

Decision support systems (DSS) powered by AI are increasingly utilized in the management of AAAs to assist with various tasks. These systems have been applied to forecast AAA growth and rupture, assess AAA morphology and fluid dynamics, and enhance image segmentation. Additionally, they have been used to create customized treatment plans and support preoperative planning [4]. It has been demonstrated that DSS is beneficial in raising patient awareness and reducing decisional conflict, which enhances informed consent and patient involvement in treatment choices. In the traditional surgeon-patient encounter, it is challenging to communicate detailed, personalized, and well-rounded information. Patients may mistakenly believe that they have no choice in the matter regarding which procedure they have or whether to have AAA surgery at all. It is important that the patient understands and is aware of the need, risks, benefits, implications, and consequences of the surgery as well as the time and healing process. Typically, there is a significant amount of complex information to convey, and the surgeon's preference may impact how options are presented to patients. These difficulties imply that patients would gain from an interactive, evidence-based decision tool [24]. In primary care practices, time constraints greatly limit a physician's capacity to provide preventative services during a normal 15-minute primary care session. This can further complicate an undiagnosed AAA, causing it to progress to fatal levels. It has been discovered that utilizing a web-based clinical DSS significantly streamlines the provision of treatment and guarantees that eligible patients receive critical preventive AAA screening. However, it is important to ensure that these systems are developed based on comprehensive information and patient preferences and to monitor final treatment decisions [25]. AI-based DSS have transformed AAA management by supporting treatment planning, assessment, and forecasting. These systems ensure prompt screening in primary care, improve patient awareness, and expedite preventive measures. A thorough understanding of patient preferences and complete information are essential for the successful implementation of DSS. Continued monitoring and refinement are crucial for optimizing patient outcomes in AAA management (Table 1).

**Table 1:** Demonstrates a summary of the utilities of AI in the enhancement of AAA management according to the sources used. AAA, abdominal aortic aneurysm; ML, machine learning; SVR, support vector regression; ANNs, artificial neural networks; BN, Bayesian networks; LOS, length of stay; PCDF, posterior cervical decompression with instrumented fusion; ARVA, augmented radiology for vascular aneurysm; HPSE, heparinase

Study	Year of publication	Method	AI model	Cohort	Results
Raffort, et al. [4]	2020	Data extraction was done, and titles and abstracts were assessed independently by two authors. 34 studies with distinct methods, goals, and research designs were found after a thorough review of the published literature.	Various AI models (not specified)	34 studies	In addition, several prognostic and predictive instruments were developed to assess patient outcomes after surgery, including death rates and complications after endovascular aneurysm repair.
Xiong, et al. [5]	2022	By employing various ML techniques to discern biomarkers distinguishing large AAA from small AAA. Validation of these biomarkers was conducted using GEO datasets. Employing CIBERSORT, evaluation of immune cell infiltration in AAA tissues alongside the exploration of correlation between biomarkers and infiltrating immune cells.	LASSO, SVM-RFE, and RF	288 differentially expressed genes	G0/G1 switch 2 (G0S2) showed strong discriminatory power as an AAA biomarker with AUC values of 0.861, 0.875, and 0.911 in GSE57691, GSE47472, and GSE7284, respectively. For large AAA, heparinase (HPSE) had AUC values of 0.669 and 0.754 in GSE57691 and GSE98278, respectively, confirmed by qRT-PCR.
Cabrera, et al. [6]	2023	The study employed the RF algorithm to analyze data from the ACS-NSQIP database spanning 2008 to 2018. It aimed to predict outcomes including LOS, readmission, reoperation, transfusion, and infection rates following elective PCDF. Independent clinical variables' significance in predicting these outcomes was evaluated using the reduction in the Gini index.	RF algorithm	12,913 patients	The ACS-NSQIP database analysis identified key patient characteristics and perioperative events for elective PCDF, such as post-operative infection, age, BMI, operative time, LOS, preoperative hematocrit, and white blood cell count. The study highlighted risk factors for reoperation, readmission, hospital LOS, transfusion needs, and post-operative infection, along with their respective AUC values.
Lee, et al. [8]	2018	To predict the future growth of AAA for individual patients, a benchmark ML method known as non-linear Kernel SVR is used. The approach taken used baseline FMD and AAA diameter as input variables.	Non-linear Kernel SVR	94 patients from OxAAA	Growth data were prospectively collected from 94 patients at 12 months and from 79 patients at 24 months. The average increase in AAA diameter was 3.4% at 12 months and 2.8% annually at 24 months. The ML algorithm accurately predicted individual AAA diameters within a 2 mm margin of error for 85% and 71% of patients at 12 and 24 months, respectively.
Karthikesaling-am, et al. [12]	2016	Aneurysm morphology was assessed pre-operatively, and endograft complications were monitored for up to 5 years post-surgery. Using ANN, researchers predicted patients' risk levels for endograft complications or mortality. Centre 1 data trained the ANN, and Centre 2 data validated it. ANN performance was evaluated using Kaplan-Meier analysis, comparing the occurrence of complications and mortality between predicted low-risk and high-risk patients.	ANN	761 patients	A total of 761 patients, with a mean age of 75 +/- 7 years, underwent EVAR, and were followed up for an average of 36+/- 20 months. The ANN, which integrated morphological features, effectively forecasted the risk of endograft complications and mortality. External validation revealed significant differences in the five-year freedom rates from aortic complications, limb complications, and mortality between low-risk and high-risk groups (p<0.001).
Monsalve-Torra A, et al. [13]	2016	The study employed various ML techniques including multilayer perceptron, radial basis function, and BNs to develop a predictive system for in-hospital mortality in patients undergoing open repair of AAA.	Multilayer perceptron, radial basis function, BNs	57 attributes from 310 cases	The examined algorithms showed over 91% accuracy, but sensitivity and specificity varied. Feature selection improved performance, particularly for RBF and BN algorithms. The highest sensitivity for death prediction was 86.8%, with specificity between 96.8% and 98.6%. Combining the three algorithms notably increased the sensitivity of mortality rate prediction.
Hadjianastassi-ou, et al. [14]	2006	The study included 1205 elective and 546 emergency AAA patients, using four independent physiological variables to predict in-hospital mortality. Both multiple regression and ANN models were developed, trained on 75% of the patient population, and tested on the remaining 25%. The evaluation included calibration, discrimination, and comparison with clinicians' estimates.	ANN	1205 elective surgery patients and 546 emergency surgery patients	In-hospital mortality rates were 9.3% for elective surgery (95% CI: 7.7%-11.1%) and 46.7% for emergency surgery (95% CI: 42.5%-51.0%). Both the ANN and statistical models outperformed clinicians' predictions in accuracy. However, only the statistical model maintained internal validity in the validation set, with good calibration (Hosmer-Lemeshow C statistic: 14.97; P=0.060) and discrimination (AUC: 0.869; 95% CI: 0.824-0.913).
Kodenko, et al. [18]	2022	The review assesses opportunistic screening models, specifically the interpretation of noncontrast CT scans including the abdominal aorta. The index test, evaluating AAA detection via AI algorithms, requires fully automatic segmentation of noncontrast CT images. Manual expert segmentation serves as the reference standard, evaluated using agreement metrics or observer expertise level.	Digital image processing algorithms, Hough’s algorithm, NN, and non-NN logical algorithm	355 cases from eight studies, with 273 cases (77%) containing AAA	The review focused on automated AAA detection or segmentation in non-contrast abdominal CT scans. The mean sensitivity value was 95%, the mean specificity value was 96.6%, and the mean accuracy value was 95.2%. However, high study heterogeneity was observed, and further research with balanced noncontrast CT datasets and adherence to reporting standards is needed to validate the high sensitivity value obtained.
Adam, et al. [20]	2021	A neural network pipeline, trained on 489 CTAs, automates the measurement process. Validation used a separate set of 62 CTAs, including controls, aneurysmal aortas, and aortic dissections, scanned before and/or after endovascular or open repair.	ARVA	Training - 489 CTAs and validation - 62 CTAs, including controls, aneurysmal aortas, and aortic dissections	The range of median absolute differences compared to expert measurements varied from 1 mm to 2 mm across all annotators, with ARVA showing a median absolute difference of 1.2 mm.
Berman, et al. [24]	2011	An interactive decision support tool, incorporating the latest outcomes data and input from surgeons and patients, was developed and piloted with AAA repair candidates. Recruited from a university vascular surgery clinic and a VA hospital, patients used the tool before meeting their surgeons. Feasibility and acceptability were gauged by participation rates, time required, assistance needed, and patient opinions. Effectiveness was evaluated by assessing changes in knowledge and decisional conflict using paired t-tests.	Decision support tool	12 patients	All approached patients (n=12) agreed to participate in the study. The tool was used for a median duration of 35 minutes (range: 25-45 minutes), and patients navigated the program with minimal technical assistance. Knowledge scores showed a significant increase from 56% to 90% (P<0.005), while decisional conflict scores decreased from 29% to 8% (P<0.04). Patients reported that the tool provided balanced information across treatment options, presented information clearly, helped them organize their thoughts, and prepared them for discussions with their surgeon.

The infancy of AI

The initial areas of focus for AI research in medicine emphasized clinical decision-making and reasoning under uncertainty, knowledge representation, and systems integration [26]. This was achieved by the development of rule-based expert systems such as MYCIN and INTERNIST, which were the foremost AI technologies to gain clearance from the FDA [27]. Such applications have also been used to diagnose patients medically, interpret results from chemical studies, and develop computer models of human behavior [28]. Today, AI in medicine is more oriented toward making predictions about future events based on deep learning or other ML methods rather than answering questions posed by scientific investigations or guiding clinical practice [29]. MYCIN and INTERNIST played a crucial role in the foundation of AI research in medicine. These rule-based expert systems, developed in the 1970s, were among the first to demonstrate the potential of AI in medical diagnosis and treatment [27]. MYCIN, for instance, was designed to treat blood infections, while INTERNIST-1 could make complex diagnoses in internal medicine [30]. MYCIN used a rule-based system and utilized production rules to represent its knowledge [31]. Similarly, INTERNIST-1 employed a combination of frames and production rules, with a focus on representing prototypical knowledge [32]. Both systems also utilized domain-independent software shells for constructing knowledge bases [33]. Additionally, INTERNIST-I incorporated properties into its knowledge representation scheme to embody essential medical information [34]. Such methods allowed the systems to represent and apply medical knowledge in their respective domains efficiently. But with this, issues arise about the scalability and adaptability of such technologies. Scalability has to be reinforced with accuracy, provided by the AI systems in diagnostics and therapeutic recommendations. Given the ethical standpoint, a question of liability arises with the AI making the decisions, leaving the attending physician liable for any mistakes. With adaptability, despite the AI making rapid decisions, an intense training regime is required for the practitioners to utilize and effectively deploy the system to aid patient health. This requires lots of time, additional training, and increased funding, which may prove to be a limiting factor in some healthcare systems with less funding [30]. These rule-based expert systems, such as INTERNIST-1 and MYCIN, faced several notable criticisms and have undergone significant development to address these issues. INTERNIST-1, for instance, struggled with accurately representing pathophysiologic causality, failing to properly attribute symptoms and disease manifestations to their causes. It also had limitations in accounting for the interdependence of symptoms and their severity, as well as in reasoning about the timing and anatomical location of symptoms. A significant concern with INTERNIST-1 was its inadequate explanation capability, which impacted user trust and acceptance. Physicians often prefer systems that can provide clear explanations for diagnostic and treatment decisions, although studies, such as those by Erdman, have indicated that the availability of explanations might sometimes lead to overconfidence in incorrect judgments. Furthermore, the need for explanations can vary depending on the user’s experience, with novices potentially requiring more detailed explanations than experienced clinicians. MYCIN, while different in approach, was among the first to emphasize the importance of explanation capabilities. It provided explanations by detailing the rules invoked for decisions, but these explanations could sometimes be superficial due to the heuristic nature of many of its rules. This approach revealed limitations in handling complex, multisystem diseases, and in providing a deep understanding of the underlying pathology. Rule-based systems, like MYCIN, face additional challenges, including the importance of the order in which rules are applied. The sequence can influence the meaning and interaction of the rules, which may not always be apparent to users. Moreover, the structure and application of rules can lead to confusion and may not be well-suited for complex diseases that involve multiple systems [33]. These early AI systems sought to assist clinical decision-making processes by offering tools for more accurate and objective decision-making in infectious disease settings [35]. Such systems were designed to increase the quality of healthcare decisions using huge amounts of data that generated useful information [36]. They also sought to enhance the abilities of doctors in gathering, understanding, and drawing conclusions from patient data, especially in advanced imaging-based clinical DSS [37]. Moreover, AI had been perceived as a potential means for identifying risk profiles, grading severity levels, and ongoing monitoring of patient’s health, although concerns were raised about data bias and misapplication [38]. The development of AI systems for clinical decision-making in the early stages faced several challenges. These challenges included the need for robust clinical evaluation, the difficulty of implementing AI systems in clinical practice, and the existence of disparity in communication between AI scientists and medical personnel [39]. Other challenges included the need for regulatory approval, interpretability, interoperability, and the use of structured data and evidence [40]. Despite these challenges, the potential for AI to enhance and complement human judgment and expertise in biomedicine is realized [29].

Challenges faced by early AI systems in the medical field have seen significant advancements, particularly with the integration of DSS powered by AI. These modern systems have greatly enhanced the management of conditions like AAAs by assisting with forecasting growth and rupture, assessing morphology and fluid dynamics, and improving image segmentation. DSS have also proven beneficial in raising patient awareness and reducing decisional conflict, which in turn enhances informed consent and patient involvement in treatment choices [24]. Traditional surgeon-patient interactions often struggle to convey the detailed, personalized, and comprehensive information required for making informed decisions. An interactive, evidence-based decision tool can help patients understand the necessity, risks, benefits, and consequences of surgery, thereby addressing the communication gaps present in traditional methods [25]. AI has further transformed AAA management by providing predictive capabilities and personalized health management plans. ML techniques, such as RF and non-linear kernel SVR, have been utilized to predict AAA growth and rupture by analyzing individual patient data, including biomarkers and baseline measurements [5,6,8,9]. These advancements allow for the creation of customized treatment plans that minimize unnecessary screenings and improve patient outcomes by focusing on lifestyle balance and planned interventions. For example, AI systems have demonstrated high sensitivity and specificity in detecting AAAs through the analysis of CT scans, MRIs, and ultrasounds, showcasing the potential of AI to enhance accuracy and efficiency in medical imaging [18,19,20,21,22,23]. 

While the potential benefits of AI techniques in managing AAAs are substantial, several critical issues and limitations require attention for a balanced perspective. First, relying on baseline FMD and AAA diameter as primary inputs for ML models may oversimplify the prediction process. AAAs are influenced by numerous factors, including genetic predisposition, lifestyle, and comorbid conditions. Focusing mainly on FMD and diameter might miss other critical variables, reducing the model's accuracy and reliability. Moreover, the effectiveness of the non-linear kernel SVR in identifying complex patterns is highly dependent on the quality and diversity of the training data. If the dataset is not representative of the broader patient population, the predictions could be biased and less generalizable. This limitation is concerning given the diverse health profiles and disease progression in AAA patients. The reported accuracy rates of 85% and 71% for predicting AAA growth within 2 mm at 12 and 24 months, respectively, are promising but indicate a significant margin of error. Underestimating AAA growth could delay necessary interventions, increasing the risk of rupture, while overestimating it could cause unnecessary anxiety, additional surveillance, and premature surgical procedures. While economic benefits through reduced premature screenings and surveillance scans are highlighted, the initial costs of implementing and maintaining sophisticated ML systems are substantial. These costs include acquiring high-quality datasets, training specialized personnel, and integrating these systems into existing healthcare infrastructure. Ongoing system updates and recalibration to ensure continued accuracy can further add to the economic burden. Another significant concern is the interpretability of ML models. Many ML techniques, especially those involving non-linear kernels, function as "black boxes," providing predictions without clear explanations. This lack of transparency can be problematic in medical contexts, where understanding the reasoning behind a prediction is crucial for clinical decision-making and gaining the trust of healthcare professionals and patients. Finally, while AI-assisted predictions are suggested to improve patient outlook and quality of life, the potential psychological impacts are not sufficiently addressed. Continuous health monitoring by an algorithm might increase patient anxiety, and prediction errors could undermine trust in medical technology. [8,9]

Medical ethics in AI

AI is on the rise with its importance in the medical field but still being premature, there are some hurdles to jump before it can be properly integrated with current medical management of AAAs. Having access to large data sets and private information leaves AI prone to data breaches for hacking, which exposes patient confidentiality. Patients may not feel comfortable putting their data at risk, which would hold back the full utilization of AI [41]. Furthermore, other studies question the reliability of this data as AI has not been incorporated for enough time to make accurate solutions for risk stratification in patients with AAA, and with natural errors occurring in all types of ML, these could lead to fatal results if not identified [42-44].

Patients' perceptions of the benefits and risks of AI in managing AAAs are influenced by various factors. It has been determined that technological, ethical, and regulatory concerns significantly contribute to the perceived risks of using AI applications in healthcare. These concerns are further influenced by the type of health condition and the nature of the clinical encounter [45]. It is reported that patients generally have minimal levels of prior information about AI but are comfortable with sharing health data with certain entities, such as the National Health Service and universities if matters regarding privacy, consent procedures, and the reidentification of anonymized healthcare data were addressed. However, they are less comfortable with sharing data with commercial organizations [46]. While some patients see the benefits of AI in healthcare, a significant portion would refuse to integrate AI-based tools into their care [47]. These findings suggest that patients' willingness to share personal data for AI-driven healthcare solutions is influenced by their perceptions of the benefits and risks of AI, as well as their trust in the entities involved in data sharing.

There are several ethical and regulatory issues associated with the use of AI in healthcare while managing AAA. Among these are problems of transparency, bias, privacy, safety, responsibility, fairness, and autonomy [48]. The legal and ethical aspects of AI healthcare applications including algorithmic transparency, privacy, and cyber security present additional complications to the integration of AI into health systems [49]. A comprehensive approach that involves policymakers, developers, healthcare providers, and patients must be put in place to tackle these issues and to achieve a robust ethical and regulatory framework for AI applications in healthcare [50]. A variety of ethical standards and regulatory frameworks have been established to govern the ethical and responsible implementation of AI in healthcare, striking a balance between fostering innovation and addressing patient safety and privacy concerns [51].

Several obstacles stand in the way of the broad use of AI technologies in the management of AAA, such as the unequal relationship between medical professionals and patients, the requirement for AI-enabled medical equipment in remote areas, and the difficulty of training doctors to use AI [52]. Even if AI can automate tasks and boost revenue, its application in the healthcare industry is hampered by the requirements for thorough clinical evaluation, comprehensible performance measures, and suitable regulation. Addressing algorithmic bias, boosting generalizability, and improving the interpretability of ML predictions are necessary for the translation of AI research into clinical practice [53]. Despite these difficulties, AI has demonstrated the potential to enhance patient care, estimate the likelihood of injuries, and interpret imaging [54].

## Conclusions

From its promising predictive computational complexities down to the genomic level, the rapid expansion and utilization of AI in the medical landscape will only continue to flourish thanks to its immense learning capabilities. Surgeons in AAA management would be able to make better decisions based on the data provided by AI, from screening to post-operative care. The application of AI holds true value across patient care as it allows an organized approach for surveillance and intervention, which will inevitably lead to reduced mortality from AAA and its complications. Supplementing the prognostic intelligence is vital for training surgeons and the incorporation of AI will only assist these clinicians in making knowledgeable decisions regarding patient care. While there may be ethical concerns surrounding patient consent, data privacy, and effects on patient-physician interactions, AI continues to hold value in the medical sector, providing potential advantages in AAA management when these concerns are handled with care.

## References

[1] Haque K, Bhargava P (2022). Abdominal aortic aneurysm. Am Fam Physician.

[2] Wanhainen A, Verzini F, Van Herzeele I (2019). Editor’s choice - European Society for Vascular Surgery (ESVs) 2019 clinical practice guidelines on the management of abdominal aorto-iliac artery aneurysms. Eur J Vasc Endovasc Surg.

[3] Rubinger L, Gazendam A, Ekhtiari S, Bhandari M (2023). Machine learning and artificial intelligence in research and healthcare. Injury.

[4] Raffort J, Adam C, Carrier M (2020). Artificial intelligence in abdominal aortic aneurysm. J Vasc Surg.

[5] Xiong T, Lv XS, Wu GJ (2022). Single-cell sequencing analysis and multiple machine learning methods identified G0S2 and HPSE as novel biomarkers for abdominal aortic aneurysm. Front Immunol.

[6] Cabrera A, Bouterse A, Nelson M (2023). Use of random forest machine learning algorithm to predict short term outcomes following posterior cervical decompression with instrumented fusion. J Clin Neurosci.

[7] Li J, Pan C, Zhang S (2018). Decoding the genomics of abdominal aortic aneurysm. Cell.

[8] Lee R, Jarchi D, Perera R, Jones A, Cassimjee I, Handa A, Clifton DA (2018). Applied machine learning for the prediction of growth of abdominal aortic aneurysm in humans. EJVES Short Rep.

[9] Shamout F, Zhu T, Clifton DA (2021). Machine learning for clinical outcome prediction. IEEE Rev Biomed Eng.

[10] Baxt WG (1995). Application of artificial neural networks to clinical medicine. Lancet.

[11] Soliman H, Elkorety M, Abouelazayem M, Girish G (2021). Short-term re-intervention of endovascular abdominal aortic aneurysm repair. Cureus.

[12] Karthikesalingam A, Attallah O, Ma X (2015). An artificial neural network stratifies the risks of reintervention and mortality after endovascular aneurysm repair; a retrospective observational study. PLoS One.

[13] Monsalve-Torra A, Ruiz-Fernandez D, Marin-Alonso O, Soriano-Payá A, Camacho-Mackenzie J, Carreño-Jaimes M (2016). Using machine learning methods for predicting inhospital mortality in patients undergoing open repair of abdominal aortic aneurysm. J Biomed Inform.

[14] Hadjianastassiou VG, Franco L, Jerez JM, Evangelou IE, Goldhill DR, Tekkis PP, Hands LJ (2006). Informed prognosis [corrected] after abdominal aortic aneurysm repair using predictive modeling techniques [corrected]. J Vasc Surg.

[15] Boufi M, Ozdemir BA (2019). Commentary: surveillance after EVAR: still room for debate. J Endovasc Ther.

[16] Schlösser FJ, Gusberg RJ, Dardik A, Lin PH, Verhagen HJ, Moll FL, Muhs BE (2009). Aneurysm rupture after EVAR: can the ultimate failure be predicted?. Eur J Vasc Endovasc Surg.

[17] Kokkinakis S, Kritsotakis EI, Lasithiotakis K (2023). Artificial intelligence in surgical risk prediction. J Clin Med.

[18] Kodenko MR, Vasilev YA, Vladzymyrskyy AV (2022). Diagnostic accuracy of AI for opportunistic screening of abdominal aortic aneurysm in CT: a systematic review and narrative synthesis. Diagnostics (Basel).

[19] Winkel DJ, Heye T, Weikert TJ, Boll DT, Stieltjes B (2019). Evaluation of an AI-based detection software for acute findings in abdominal computed tomography scans: toward an automated work list prioritization of routine CT examinations. Invest Radiol.

[20] Adam C, Fabre D, Mougin J (2021). Pre-surgical and post-surgical aortic aneurysm maximum diameter measurement: full automation by artificial intelligence. Eur J Vasc Endovasc Surg.

[21] Abbas A, Smith A, Cecelja M, Waltham M (2012). Assessment of the accuracy of aortascan for detection of Abdominal Aortic Aneurysm (AAA). Eur J Vasc Endovasc Surg.

[22] Garvin T, Kimbleton S (2021). Artificial intelligence as Ally in hazard analysis. Process Saf Prog.

[23] Aboyans V, Bataille V, Bliscaux P (2014). Effectiveness of screening for abdominal aortic aneurysm during echocardiography. Am J Cardiol.

[24] Berman L, Curry L, Goldberg C, Gusberg R, Fraenkel L (2011). Pilot testing of a decision support tool for patients with abdominal aortic aneurysms. J Vasc Surg.

[25] Knops AM, Goossens A, Ubbink DT, Legemate DA (2011). Regarding "pilot testing of a decision support tool for patients with abdominal aortic aneurysms". J Vasc Surg.

[26] Patel VL, Shortliffe EH, Stefanelli M, Szolovits P, Berthold MR, Bellazzi R, Abu-Hanna A (2009). The coming of age of artificial intelligence in medicine. Artif Intell Med.

[27] Chang AC (2020). History of artificial intelligence in medicine. Intelligence-Based Medicine.

[28] Coles LS (1977). The application of artificial intelligence to medicine. Futures.

[29] Kulikowski CA (2019). Beginnings of artificial intelligence in medicine (AIM): computational artifice assisting scientific inquiry and clinical art - with reflections on present aim challenges. Yearb Med Inform.

[30] Jaiswal R, Sapra RL, Jha GK, Nundy S (2020). Artificial intelligence in medical diagnosis. J Curr Med Res Pract.

[31] Davis R, Buchanan B, Shortliffe E (1977). Production rules as a representation for a knowledge-based consultation program. Artif Intell.

[32] Aikins JS (1983). Prototypical knowledge for expert systems. Artif Intell.

[33] Perry CA (1990). Knowledge bases in medicine: a review. Bull Med Libr Assoc.

[34] Masarie FE, Miller RA, Myers JD (1985). INTERNIST-I properties: representing common sense and good medical practice in a computerized medical knowledge base. Comput Biomed Res.

[35] Garcia-Vidal C, Sanjuan G, Puerta-Alcalde P, Moreno-García E, Soriano A (2019). Artificial intelligence to support clinical decision-making processes. EBioMedicine.

[36] Walczak S (2018). The role of artificial intelligence in clinical decision support systems and a classification framework. Int J Comput Clin Pract.

[37] Shaikh F, Dehmeshki J, Bisdas S, Roettger-Dupont D, Kubassova O, Aziz M, Awan O (2021). Artificial intelligence-based clinical decision support systems using advanced medical imaging and radiomics. Curr Probl Diagn Radiol.

[38] Giordano C, Brennan M, Mohamed B, Rashidi P, Modave F, Tighe P (2021). Accessing artificial intelligence for clinical decision-making. Front Digit Health.

[39] Rathinam AK, Lee Y, Chek Ling DN, Singh R, Selvaratnam L, Pamidi N (2021). Artificial intelligence in medicine: a review of challenges in implementation and disparity. IEEE.

[40] Varghese J (2020). Artificial intelligence in medicine: chances and challenges for wide clinical adoption. Visc Med.

[41] Farhud DD, Zokaei S (2021). Ethical issues of artificial intelligence in medicine and healthcare. Iran J Public Health.

[42] Haller SJ, Azarbal AF, Rugonyi S (2020). Predictors of abdominal aortic aneurysm risks. Bioengineering (Basel).

[43] Keskinbora KH (2019). Medical ethics considerations on artificial intelligence. J Clin Neurosci.

[44] Tang L, Li J, Fantus S (2023). Medical artificial intelligence ethics: a systematic review of empirical studies. Digit Health.

[45] Esmaeilzadeh P (2020). Use of AI-based tools for healthcare purposes: a survey study from consumers' perspectives. BMC Med Inform Decis Mak.

[46] Aggarwal R, Farag S, Martin G, Ashrafian H, Darzi A (2021). Patient perceptions on data sharing and applying artificial intelligence to health care data: cross-sectional survey. J Med Internet Res.

[47] Tran VT, Riveros C, Ravaud P (2019). Patients' views of wearable devices and AI in healthcare: findings from the ComPaRe e-cohort. NPJ Digit Med.

[48] Pasricha S (2022). AI ethics in smart healthcare. IEEE Consumer Electronics Magazine.

[49] Naik N, Hameed BM, Shetty DK (2022). Legal and ethical consideration in artificial intelligence in healthcare: who takes responsibility?. Front Surg.

[50] Prakash S, Balaji JN, Joshi A, Surapaneni KM (2022). Ethical conundrums in the application of artificial intelligence (AI) in healthcare-a scoping review of reviews. J Pers Med.

[51] Corrêa NK, Galvão C, Santos JW (2023). Worldwide AI ethics: a review of 200 guidelines and recommendations for AI governance. Patterns (N Y).

[52] Nizam V, Aslekar A (2021). Challenges of applying AI in healthcare in India. J Pharm Res Int.

[53] Kelly CJ, Karthikesalingam A, Suleyman M, Corrado G, King D (2019). Key challenges for delivering clinical impact with artificial intelligence. BMC Med.

[54] Ramkumar PN, Kunze KN, Haeberle HS, Karnuta JM, Luu BC, Nwachukwu BU, Williams RJ (2021). Clinical and research medical applications of artificial intelligence. Arthroscopy.

